# Wearable, cuffless, and portable devices for blood pressure monitoring (2015–2025): a scoping review of technologies, calibration, clinical validation, and regulatory frameworks

**DOI:** 10.3389/fdgth.2026.1870156

**Published:** 2026-08-12

**Authors:** Juan Carlos Bustamante-Rodríguez, Jhosmer Ballena-Caicedo, Julio César Bautista-Zuta, Víctor Juan Vera-Ponce

**Affiliations:** Facultad de Medicina (FAMED), Universidad Nacional Toribio Rodríguez de Mendoza de Amazonas (UNTRM), Chachapoyas, Peru

**Keywords:** ambulatory blood pressure monitoring, blood pressure, cuffless devices, hypertension, photoplethysmography, scoping review, validation study, wearable electronic devices

## Abstract

**Background:**

Out-of-office blood pressure (BP) monitoring is essential for diagnosing and controlling hypertension. Between 2015 and 2025, wearable devices and portable cuffless platforms emerged that estimate BP without a conventional brachial cuff; their evaluation is complicated by technological heterogeneity, calibration, sensitivity to dynamic conditions, and recent device-specific standards.

**Objective:**

To map the evidence published between 2015 and 2025 on wearable and portable cuffless devices for BP measurement or estimation, describing form factors, technologies, calibration/recalibration, validation, populations, settings, and standards or regulatory frameworks.

**Methods:**

A scoping review was conducted in accordance with PRISMA-ScR and the Joanna Briggs Institute, using the PCC framework. Sources were searched in PubMed/MEDLINE, Scopus, Web of Science, and Embase, supplemented by device-directed searches and citation chasing. Extraction captured population, device, technology, setting, calibration, standard/protocol, comparator, metrics, limitations, conflicts, and funding.

**Results:**

Sixty-three sources were included: 7 standards/consensus/regulatory documents, 1 case study, 6 reviews, and 49 primary studies. Primary evidence covered watches with miniaturized cuffs, wearable or portable cuffless devices, and rings/finger devices. Oscillometric wrist watches were methodologically closer to conventional automated monitors, although limited by positioning requirements, reading errors, and scarce ambulatory evidence. Cuffless devices relied on PPG/PWA, PTT/PAT, tonometry, or multimodal approaches, with calibration/recalibration and proprietary algorithms. Critical gaps persisted in post-calibration stability, dynamic conditions, sleep, treatment, arrhythmias, and special populations.

**Conclusions:**

The 2015–2025 ecosystem shows rapid progress and a transition toward cuffless-specific validation. However, clinical utility depends on intended use, population, calibration/recalibration, and setting. Performance should not be extrapolated across technologies, populations, or contexts.

## 1 Introduction

Accurate and repeated blood pressure (BP) measurement is a core component of hypertension diagnosis, risk stratification, and therapeutic follow-up. Out-of-office measurement—including home blood pressure monitoring and 24-h ambulatory blood pressure monitoring (ABPM)—helps confirm diagnoses, identify white-coat or masked hypertension, and characterize circadian patterns such as nocturnal dipping (1, 2). Nevertheless, cuff-based devices remain intermittent, depend on correct technique, and may be uncomfortable for frequent or prolonged measurements (3).

Over the past decade, wearable and portable platforms have been developed to estimate BP using watches, wristbands, patches, rings, finger sensors, and pocket devices. These technologies include miniaturized cuffs, photoplethysmography (PPG), pulse-wave analysis, pulse transit time or pulse arrival time (PTT/PAT), tonometry, multimodal signals, and machine-learning models (4, 5). Their clinical promise is to increase measurement frequency, reduce user burden, and approximate BP under real-life conditions; however, that promise does not equate to automatic clinical validity (6).

The evaluation of these devices cannot simply transpose standards designed for conventional sphygmomanometers. Recent scientific statements and recommendations emphasize that cuffless devices estimate BP indirectly, with performance conditioned by calibration, posture, sensor height relative to the heart, motion, peripheral perfusion, temperature, algorithm, and intended use (7–9). In addition, ISO 81060-3 and IEEE 1708a incorporate approaches for continuous or cuffless technologies, but gaps remain regarding post-calibration stability, dynamic conditions, arrhythmias, special populations, and algorithmic transparency (10, 11).

In this context, a scoping review is appropriate because the field combines clinical evidence, biomedical engineering, standards documents, regulatory studies, and reviews with heterogeneous methods. The gap is not merely whether devices work, but which technologies have been evaluated, against which comparators, with what calibration, in which populations, and which gaps prevent robust clinical inference. Therefore, the overall objective of this scoping review was to map the evidence published between 2015 and 2025 on wearable and portable cuffless devices for BP measurement or estimation, describing form factors, technologies, calibration/recalibration, validation, populations, settings, and regulatory frameworks. Specifically, we aimed to: (i) inventory and classify devices by form factor and measurement principle; (ii) describe calibration requirements and stability; (iii) characterize reference comparators, applied protocols, and reported metrics; (iv) map the populations and settings evaluated; and (v) identify gaps for future research and responsible clinical adoption.

## 2 Materials and methods

### 2.1 Design and reporting guidance

We conducted a scoping review in accordance with the PRISMA extension for scoping reviews (PRISMA-ScR) and the methodological guidance of the Joanna Briggs Institute (12, 13). The protocol was not formally registered; the data-extraction table, complete search strategies, and operational criteria are provided in the Supplementary Material. The completed PRISMA-ScR checklist is provided in Supplementary Material 2. No formal risk-of-bias or certainty-of-evidence assessment was performed because the objective was to map the field and its gaps, not to estimate a pooled effect or issue a quantitative clinical recommendation.

Sources were classified into three strata: primary studies in humans, secondary evidence—systematic reviews, meta-analyses, or narrative reviews—and standards/regulatory documents. Standards or regulatory documents were included only when they provided explicit validation criteria, intended-use information, comparators, or performance requirements; they were not counted as primary studies and were not used to infer device clinical performance. Accordingly, clinical-performance statements were derived from primary human studies, whereas standards, consensus statements, and regulatory documents were synthesized as methodological context defining validation requirements, intended-use constraints, comparators, and reporting gaps.

### 2.2 PCC framework and eligibility criteria

We used the Population–Concept–Context (PCC) framework. The population included human studies of any age, both in the general population and in clinical settings (for example, hypertension, pregnancy, Parkinson disease, stroke, postoperative care, ICU, or catheterization), without restriction by sex, country, or level of care. We included sources published between January 1, 2015, and December 31, 2025, in any language, provided they reported sufficient information to extract at least the device type and a BP outcome or related performance metric.

The concept included: (a) wearable devices with miniaturized cuffs, such as oscillometric wrist watches; and (b) wearable or portable cuffless devices based on PPG/PWA, PTT/PAT, ECG + PPG, tonometry, finger sensors, ultrasonography, magnetoplethysmography, seismocardiography, or multimodal approaches reporting SBP and/or DBP. The context covered clinical or laboratory validations, ambulatory or real-life evaluations, comparisons with ABPM, home measurement, and hospital settings, including comparisons with invasive references. We excluded animal studies, simulations without human data, publications outside the study period, sources without BP outcomes, and articles with insufficient information for minimum extraction.

### 2.3 Information sources and search strategy

We searched PubMed/MEDLINE, Scopus, Web of Science Core Collection, and Embase (Elsevier) between February 2 and 15, 2026, applying publication-year filters to retrieve literature published between 2015 and 2025. The strategies combined condition/outcome terms (blood pressure, hypertension), technology and form-factor terms (cuffless, wearable, smartwatch, wristband, ring, patch, finger, PPG, PTT, PAT, tonometry, pulse wave analysis), and evaluation terms (validation, accuracy, agreement, comparison, performance). The complete strategies are presented in Supplementary Appendix 1.

To increase sensitivity, we conducted targeted searches by commercial name, project name, or technology family, including HeartGuide, HEM-6410T, HUAWEI WATCH D/D2, Aktiia/Hilo, SOMNOtouch-NIBP, SeismoWatch, CART-I Plus, Freescan, Checkme, Biobeat, CareTaker, VitalStream, Aurora-BP, Microsoft Aurora, BPro, Samsung Galaxy Watch, SM-R850, and KC08. Backward and forward citation chasing was also performed from guidelines, reviews, and pivotal device studies.

### 2.4 Reference management and source selection

Records were exported from each database in compatible formats and consolidated into a single library. Duplicates were removed using combined criteria for title, authors, year, DOI/PMID, and journal, supplemented by manual review when metadata discrepancies were present. The deduplicated set was screened by title/abstract and then by full text. Discrepancies were resolved by consensus and, when necessary, by a third evaluator. At full-text review, predefined exclusion reasons were recorded: absence of BP or an equivalent metric, device not wearable or portable cuffless, absence of human data, publication outside the period, or insufficient reporting.

### 2.5 Data extraction and synthesis

A standardized extraction form was developed and iteratively piloted in a subset of sources. For each source, we extracted: population, device, form factor, technological principle, measurement setting, calibration/recalibration, standard or protocol applied, reference comparator, metrics, limitations, conflicts of interest, and funding. Cells marked “NR” indicate information not reported; “NA” indicates that the parameter was not applicable. Supplementary Table S1 contains the complete extraction.

The synthesis was descriptive, consistent with the objective of a scoping review. It was organized by evidence strata and technology categories: miniaturized cuffs, wearable or portable cuffless devices, rings/finger devices, standards/regulatory sources, and reviews. Because of the heterogeneity of comparators, measurement conditions, and metrics, no meta-analysis was planned.

## 3 Results

### 3.1 Source selection

**Figure 1:**
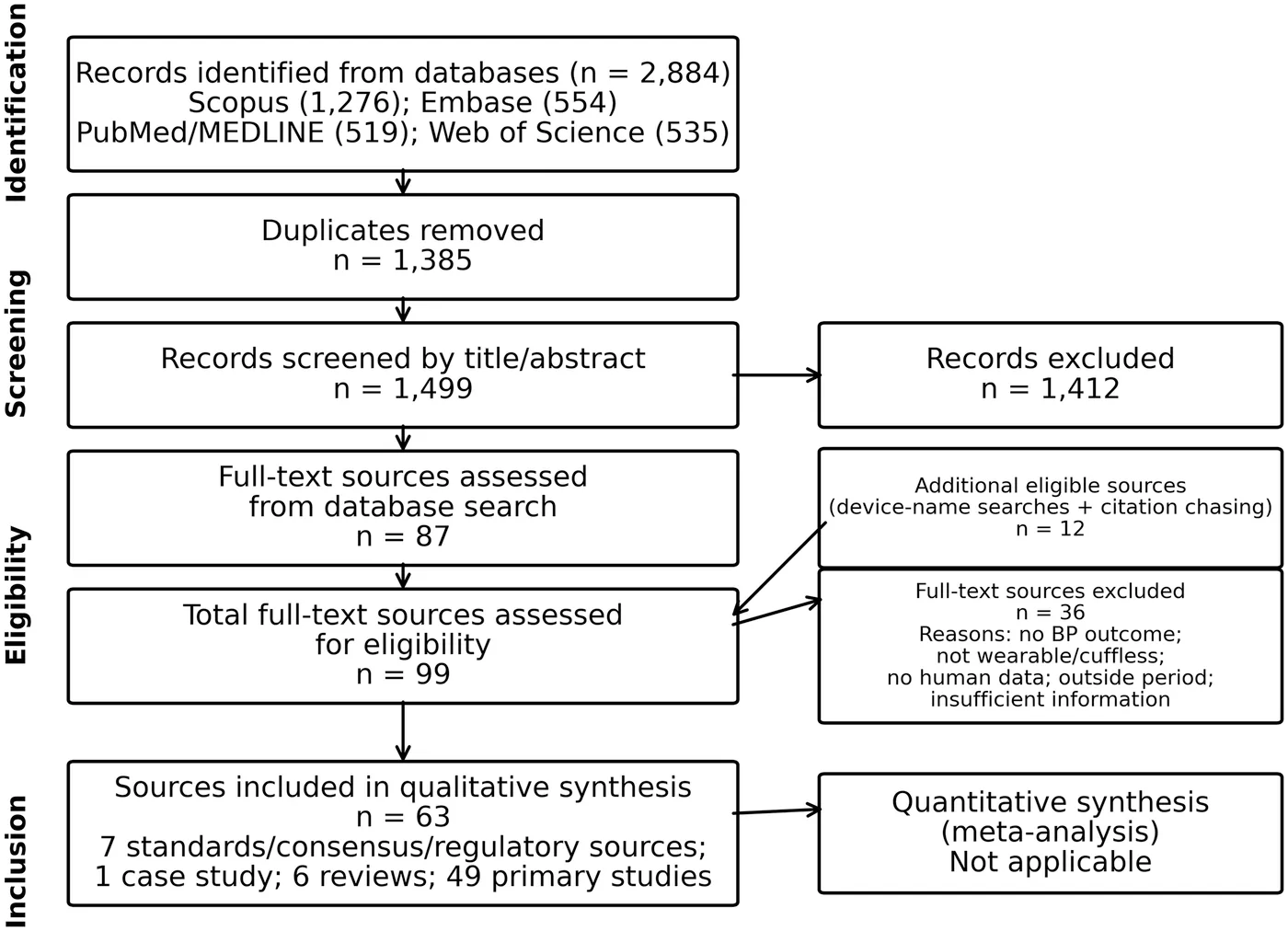
Updated PRISMA-ScR flow diagram for the source-selection process. The diagram displays the number of records identified across PubMed/MEDLINE, Scopus, Web of Science, and Embase; the number removed as duplicates; the number screened by title/abstract and by full text; the additional records identified through device-name–directed searches and citation chasing; and the final number of sources included in the qualitative synthesis (*n* = 63).

We identified 2,884 records in bibliographic databases: Scopus (*n* = 1,276), Embase (*n* = 554), PubMed/MEDLINE (*n* = 519), and Web of Science (*n* = 535). After removing 1,385 duplicates, 1,499 records were screened by title/abstract and 1,412 were excluded. Eighty-seven sources were assessed at full text through the bibliographic route. Device-name–directed searching and citation chasing identified 12 additional eligible sources. Overall, 99 sources were assessed at full text, 36 were excluded, and 63 were included in the qualitative synthesis. No quantitative synthesis or meta-analysis was performed (Figure 1).

### 3.2 General characteristics of the included evidence

The 63 included sources comprised 7 standards, consensus, or regulatory documents (7–11, 14, 15), 6 systematic, narrative, or interpretive reviews (16–21), 1 case study or self-assessment (22), and 49 primary human studies (23–71). Primary evidence included 8 studies of watches with miniaturized cuffs (23–30), 33 studies of wearable or portable cuffless devices (31–63), and 8 studies of rings or finger devices (64–71) (Table 1). Detailed extraction is presented in Supplementary Table S1, and the device map in Supplementary Material S2.

**Table 1:** Summary of the included evidence base.

Evidence stratum/category	*n*	Examples	Methodological interpretation
Standards/consensus/regulatory documents	7	AAMI/ESH/ISO, IEEE 1708a, ISO 81060-3, ESH recommendations, AHA, FDA 510(k)	Define validation requirements, comparators, stability, and intended use; they are not primary evidence of clinical performance.
Reviews	6	Systematic reviews, meta-analyses, narrative reviews, and interpretive reviews, including the Microsoft Aurora Project commentary	Describe technological heterogeneity, lack of independent validation, and the need for specific protocols; interpretive sources contextualize large device-specific projects.
Case study/self-assessment	1	Individual comparison of cuffless devices	Useful for illustrating real-world inconsistencies; not generalizable.
Watches with miniaturized cuffs	8	Omron HeartGuide/HEM-6410T; HUAWEI WATCH D/D2	Intermittent oscillometric technology; should not be conceptually grouped with optical cuffless devices.
Cuffless wearables and portable devices	33	Samsung, Aktiia, Biobeat, Aurora-BP, SOMNOtouch-NIBP, Checkme, Freescan, BPro, SeismoWatch, KC08, prototypes	Heterogeneous evidence; performance depends on calibration, position, motion, perfusion, algorithm, and context.
Rings/finger devices	8	CART-I Plus; CareTaker/VitalStream	Evidence now includes perioperative, catheterization, ICU, emergency department, and pediatric surgical settings; external validation under dynamic conditions and transparent artifact handling remain essential.

### 3.3 Devices with miniaturized cuffs

Watch-type oscillometric devices with miniaturized cuffs retain a measurement principle based on local occlusion with a wrist cuff and therefore should be distinguished from cuffless devices. We identified 8 primary studies published between 2019 and 2025 on Omron and Huawei devices (23–30). Most were conducted in clinical or controlled settings; only a subset evaluated ambulatory conditions or comparisons with ABPM. These devices were validated mainly against traditional standards for automated sphygmomanometers, such as ANSI/AAMI/ISO 81060-2 or the Universal Standard.

In this category, keeping the watch at heart level is a positioning and measurement requirement, not individual algorithmic calibration. This distinction matters because conflating position with calibration changes the methodological reading of the studies. The predominant metrics were mean difference, standard deviation, ISO criteria 1 and 2, Bland–Altman analysis, correlation or concordance coefficients, and, less frequently, mixed-effects models. The most frequent limitations were sensitivity to wrist position, order effects in consecutive same-arm measurements, errors or invalid readings, small samples, exclusions based on wrist circumference, and validation on a single wrist. Funding or links with manufacturers were identified in several studies.

### 3.4 Wearable and portable cuffless devices

Cuffless devices were evaluated in 33 primary studies and one case study (22, 31–63). This category included smartwatches, optical wristbands, patches, pocket devices, radial tonometry, multimodal sensors, magnetoplethysmography, seismocardiography, ultrasonography, and machine-learning models. Unlike watches with miniaturized cuffs, these systems estimate BP indirectly from physiological signals and algorithms; therefore, their performance depends on calibration, temporal stability, signal quality, motion, posture, peripheral perfusion, and intended use.

Calibration was heterogeneous. Some studies used initial cuff measurements, others incorporated age, sex, weight, height, or individual profiles, and a smaller group reported calibration-free approaches or did not describe the method. Comparators included auscultation, validated oscillometric devices, ABPM, home measurement, and invasive blood pressure. The most frequent metrics were Bland–Altman analysis, mean difference, standard deviation, limits of agreement, correlation, mean absolute error, sensitivity/specificity, and classification analyses. Applied standards varied: ANSI/AAMI/ISO, ESH-IP, IEEE 1708, ISO 81060-2, ESH recommendations, FDA regulatory criteria, or national standards.

The incorporation of additional device-directed sources modified the evidence map. Two Biobeat studies evaluated a cuffless PPG device in repeated measurements against a sphygmomanometer and in 24-h ABPM, contributing evidence for ambulatory use while noting limitations due to sample size and underrepresentation of hypertensive participants or extreme subgroups (44, 45). Ahn et al. evaluated a Samsung SM-R850 in patients with Parkinson disease, a previously underrepresented population because of the potential problem of involuntary movement (35). Tan et al. provided an independent evaluation of Aktiia for tracking changes, showing limitations in capturing nocturnal dipping and medication-induced changes (41). López-Ortiz et al. evaluated the KC08 smartwatch and concluded that it was not suitable for daily clinical BP practice, adding relevant negative evidence for low-cost commercial devices (62).

The Microsoft Aurora-BP project adds a large independent dataset to this evidence landscape. Mieloszyk et al. evaluated wearable tonometry, photoplethysmography, electrocardiography, and accelerometry for cuffless BP estimation in controlled and ambulatory settings (63). The study showed that reference type, posture relative to calibration, and controlled vs. ambulatory testing materially affected BP-estimation error. Tonometry-derived features appeared physiologically informative when signal quality was available, but the findings did not support a simple technology-wide endorsement. The accompanying interpretation by Mukkamala et al. emphasized that Aurora should temper reliance on static validation and reinforce intended-use testing, post-calibration stability, and real-world evaluation (21).

Overall, the cuffless evidence does not support a single conclusion by technology family. Some devices met agreement criteria under specific conditions, whereas others failed in ambulatory settings, change tracking, or real-world practice. This pattern reinforces that validation must be judged by device, algorithm, population, comparator, calibration, and use setting, not by the generic label “wearable” or “cuffless.”

### 3.5 Rings and finger devices

We identified 8 primary studies on rings or finger-sensor devices (64–71). Devices included CART-I Plus and CareTaker/VitalStream platforms. CART-I Plus studies evaluated finger PPG-based cuffless BP in clinical and out-of-office contexts using auscultatory or ABPM comparators. CareTaker/VitalStream studies broadened the evidence base by evaluating pulse decomposition analysis in perioperative, catheterization, ICU, emergency department, and pediatric surgical settings, including comparisons with invasive intra-arterial, central aortic, brachial cuff, or ABPM references. The added CareTaker/VitalStream evidence is clinically informative because it includes both favorable validation studies and pragmatic negative evaluations. Gratz et al. reported perioperative CareTaker performance against invasive intra-arterial pressure in adult abdominal surgery (67, 68), Kwon et al. evaluated beat-to-beat tracking against invasive central aortic pressure (69), and Boretsky et al. evaluated VitalStream in pediatric surgery (70). Conversely, Hamilton et al. directly assessed VitalStream in emergency department and ICU settings and found that all direct comparisons failed to meet AAMI 2008 and AAMI/ESH/ISO 2019 consensus criteria, with standard deviations exceeding 8 mmHg and fewer than 85% of paired differences falling within 10 mmHg of the reference (71). This discrepancy across studies appears partly related to setting, reference type, calibration approach, movement artifact, signal-quality exclusion, and postprocessing. Therefore, the CareTaker/VitalStream evidence should not be summarized as uniformly positive or negative; it should be interpreted as device-family evidence that is strongly conditioned by clinical setting, artifact handling, and intended use.

### 3.6 Standards, regulatory sources, and reviews

The included standards and consensus documents establish that validation of cuffless devices should consider static, positional, change-tracking, sleep/wake, exercise, and recalibration tests according to the technology and intended use (7–11, 14, 15). Regulatory authorization or equivalence should not be interpreted as universal clinical validity; its scope depends on the product, indication, population, and comparators evaluated. The included reviews and interpretive articles agreed on methodological heterogeneity, the need for independent validation, and the risk of extrapolating results across technologies (16–21).

## 4 Discussion

This updated scoping review maps 63 sources on wearable, cuffless, and portable BP devices between 2015 and 2025. The central finding is that the field is growing rapidly, but evidence is not interchangeable across devices. Watches with miniaturized cuffs, calibrated cuffless smartwatches, persistent optical wristbands, patches, rings, finger devices, and large multimodal projects represent distinct metrological architectures, with distinct validation requirements and sources of error.

Algorithmically, the field is moving from single-surrogate approaches, particularly PTT/PAT or PWV-based estimation, toward device-specific multimodal and data-driven models that combine PPG/PWA morphology, ECG timing, demographic covariates, accelerometry, posture information, tonometry, and proprietary cloud-based algorithms. This transition should not be interpreted as evidence that deep learning is intrinsically superior. Greater model flexibility may improve apparent performance under narrow calibration or training conditions, but it also increases dependence on training-set representativeness, calibration leakage, algorithm-version control, and external validation. The Aurora-BP evidence is informative in this respect: physiologically richer signals such as tonometry may improve information content when signal quality is adequate, but performance remains strongly affected by reference type, posture, calibration, and whether testing is controlled or ambulatory (21, 63).

The first methodological implication is to separate oscillometric watches with miniaturized cuffs from cuffless devices. Although both may have a watch form factor, the former preserves an oscillometric local-occlusion mechanism, whereas the latter infers BP from indirect physiological signals. Grouping them under the same label dilutes essential issues: wrist position and height relative to the heart for the former; calibration, algorithmic stability, peripheral perfusion, and signal artifacts for the latter (7–11, 72–74).

Calibration emerges as a critical axis. In devices with miniaturized cuffs, watch height relative to the heart is a measurement condition. In cuffless devices, calibration may be individual, demographic, cuff-based, baseline-profile based, or reportedly absent. The uncomfortable—but methodologically unavoidable—point is that acceptable baseline validation does not demonstrate longitudinal stability. The added evidence on change tracking, nocturnal dipping, pragmatic acute-care testing, and the Aurora-BP project shows that detecting average values is not equivalent to capturing clinically relevant trajectories (21, 41, 63, 71).

Regulatory clearance or equivalence should be interpreted as evidence that a device met the requirements of a defined submission, not as proof that it can track BP trajectories under all real-life conditions. For cuffless devices initialized or calibrated against cuff measurements, high cross-sectional correlation with a cuff reference may partly reflect calibration at similar pressure ranges, regression to the population BP distribution, and the limited dynamic challenge imposed by static seated protocols. Therefore, correlation or immediate post-calibration agreement should not be interpreted as evidence of faithful within-person BP-change tracking. Validation should separately report static accuracy, post-calibration drift, recalibration intervals, dynamic-condition performance, sleep/wake performance, treatment-response tracking, and within-person BP-change detection against an intended-use-appropriate reference. The term “calibration-free” should therefore be used operationally rather than literally. In this literature, it usually means that no user-entered cuff BP value is required at initialization; it does not mean that the device is exempt from longitudinal verification. Calibration-free algorithms still require external validation across time, BP ranges, sensor placement, vascular phenotypes, software versions, and use conditions.

The greater concentration of studies in clinical settings probably reflects the need to control comparators, posture, measurement sequence, BP distribution, and signal acquisition conditions. However, performance under controlled conditions does not automatically imply ambulatory utility. Real-world measurements introduce motion, temperature, perfusion variation, sleep, activity, user errors, therapeutic changes, and synchronization artifacts. The included evidence shows both positive and negative results precisely because these domains are not ancillary: they are the actual environment of use.

Evidence in special populations remains insufficient. The inclusion of patients with Parkinson disease and stroke provides useful signals, but the studies remain small or controlled at rest (35, 36). Arrhythmias, obesity, peripheral vascular disease, variation by skin tone, pregnancy, older age, pediatric surgical patients, ICU and emergency populations, and vasopressor use remain underrepresented or setting-specific. These signal-vulnerable populations should not be excluded from the evidence map; rather, they should be analyzed as stress tests of external validity and reported separately from general-population accuracy claims. For a device that depends on optical or peripheral hemodynamic signals, assuming generalizability without data would be technological optimism rather than clinical inference.

A substantial dependence on manufacturer funding or participation also persists. Conflicts of interest do not automatically invalidate a study; that would be an overly simplistic interpretation. However, they do require distinguishing sponsored validation, independent validation, regulatory validation, and real-world performance evaluation. In this review, several devices had favorable initial evidence alongside independent studies revealing limitations in dynamic or longitudinal settings. This tension should be preserved in interpretation rather than obscured for narrative convenience (75, 76).

Comparator heterogeneity should be interpreted in relation to intended use rather than as a single hierarchy of validity. No universal gold standard applies across all wearable and cuffless BP scenarios. Double-observer auscultation remains the conventional reference for intermittent static validation; invasive arterial pressure is the most direct comparator in ICU, perioperative, and catheterization contexts; and validated ABPM or HBPM devices are pragmatic comparators when the intended use is out-of-office monitoring. Therefore, future studies should justify the comparator according to the clinical claim being made, rather than reporting generic agreement against any available cuff device.

From a clinical perspective, these devices should not replace validated cuff-based measurements when formal diagnosis or therapeutic adjustment is required (77), unless the device, population, and setting match a robust validation and its intended use. Responsible adoption requires specifying the device, algorithm version, calibration method, recalibration interval, comparator, invalid-reading criteria, and context of use. The claim that a device “measures BP” is too crude for a field in which technical detail determines validity.

A practical reporting framework for future cuffless BP studies should be organized by intended use. Static validation should report mean error or bias, SD, limits of agreement, MAE/MAD or RMSE, and the proportion of readings within 5, 10, and 15 mmHg, with BP distribution across clinically relevant ranges. Ambulatory or continuous-use validation should additionally report synchronization rules, invalid-reading rates, posture/activity/sleep labels, day/night performance, within-person change tracking, treatment-response performance, time since calibration, recalibration triggers, software or algorithm version, and subgroup performance by age, sex, BP range, arrhythmia, obesity, skin tone or peripheral perfusion, pregnancy, and relevant comorbidities. Until cuffless-specific evidence is more mature, acceptable performance should be judged against recognized device-specific standards and intended-use tests rather than by correlation alone.

As a scoping review, this work did not estimate pooled effects or perform a formal risk-of-bias assessment; therefore, the synthesis describes heterogeneity and gaps but does not allow quantitative ranking of the accuracy of each technology. The joint inclusion of primary studies, reviews, standards, and regulatory documents increases the breadth of the map, but it requires caution: these sources do not provide the same level of evidence on clinical performance. Finally, although four databases, targeted searches, and citation chasing were used, the variable terminology of the field may have missed studies indexed under unanticipated commercial names or technology descriptors.

Another limitation is that some regulatory documents or standards do not provide complete primary clinical data, and some included studies did not transparently report calibration, algorithms, invalid-reading rates, or BP distribution. These omissions limit comparability across devices and reinforce the need for intended-use–specific standards. Sources published after 2025 were not included in the synthesis; when they are mentioned in future versions of the manuscript, they should be explicitly declared as context after the inclusion period.

## 5 Conclusions

The evidence published between 2015 and 2025 on wearable, cuffless, and portable BP devices shows a rapidly evolving field, but one constrained by technological heterogeneity, calibration dependence, variable performance under dynamic conditions, and limited independent validation in special populations. For clinical use, devices should be interpreted strictly within their intended use, evaluated population, calibration protocol, and applied validation standard. The mapped evidence does not justify extrapolating the performance of one device to another technology, population, or use setting, nor replacing validated cuff-based measurements when diagnosis or therapeutic adjustment is required. Future research should prioritize independent, multicenter, ambulatory validations with longitudinal follow-up, recalibration testing, and explicit assessment of invalid readings, arrhythmias, motion, and clinically relevant subgroups.

## Data Availability

All data generated or extracted during this scoping review are included in the article and its Supplementary Material. The source-level extraction, device map, and database search strategies are provided in Supplementary Material 1, and the completed PRISMA-ScR checklist is provided in Supplementary Material 2. Further inquiries can be directed to the corresponding author.
