# Supplementary material for: Wearable, cuffless, and portable devices for blood pressure monitoring (2015–2025): a scoping review of technologies, calibration, clinical validation, and regulatory frameworks
Source: Front Digit Health. 2026 Aug 12;8:1870156. doi: 10.3389/fdgth.2026.1870156 (PMC13506816; doi:10.3389/fdgth.2026.1870156)
Supplement: Supplementary file 1 [file Supplementaryfile1.docx]

Supplementary Material 1

*Wearable, cuffless, and portable devices for blood pressure monitoring (2015–2025): a scoping review of technologies, calibration, clinical validation, and regulatory frameworks*

This supplementary file accompanies the main manuscript and includes: (i) Supplementary Table S1, full data extraction of the 63 included sources; (ii) Supplementary Material S2, a device map summarizing technologies, evidence, and gaps; and (iii) Appendix 1, the complete database search strategies for PubMed/MEDLINE, Scopus, Embase, and Web of Science, together with complementary device-directed searches. The completed PRISMA-ScR checklist is provided separately as Supplementary Material 2.

**Supplementary Table S1.** Included sources and data extraction.

| **Evidence type** | **Author** | **Year** | **Population** | **Concept / device** | **Setting** | **Calibration** | **Protocol / Standard** | **Comparator / Reference** | **Key metrics** | **Limitations** | **Conflicts of interest** | **Funding** |
| --- | --- | --- | --- | --- | --- | --- | --- | --- | --- | --- | --- | --- |
| Consensus | Stergiou GS et al. | 2018 | NA | BP device validation | Clinical validation protocol | NR | Universal Standard (AAMI/ESH/ISO) | Auscultatory standard | Mean difference and SD; Bland–Altman | Not cuffless-specific; preliminary document | Multiple authors conducted studies for manufacturers | None |
| Standard | IEEE Std 1708a-2019 | 2019 | NA | Wearable cuffless BP evaluation | Static, pressure-change and post-calibration evaluation | Patient parameters: height, weight, sex, age | IEEE 1708a-2019 | Mercury sphygmomanometer reference | MAD, MAPD, MD, SD, CP5, CP10, CP15 | Limited assessment outside sitting/static conditions | NR | IEEE Standards Association |
| Standard | ISO 81060-3:2022 | 2022 | NA | Continuous non-invasive BP | Clinical investigation | NA | ISO 81060-3:2022 | Invasive BP monitoring equipment | MD, SD, RMSD | Excludes usability, visualization and motion artifacts; population constrained by invasive reference | NR | NR |
| Consensus | Stergiou GS et al. | 2022 | NA | Cuffless BP devices | Technology and validation statement | Usually require individual cuff calibration | IEEE/AAMI/ESH/ISO | Manual auscultatory or intra-arterial reference | Mean error, SD, Bland–Altman | NR | Extensive author links with manufacturers | NR |
| Consensus | Stergiou GS et al. | 2023 | NA | Intermittent cuffless BP devices | Static, position, treatment, sleep/exercise and recalibration tests | Cuff, demographic or calibration-free depending on device | ESH recommendations | Double-observer auscultation or 24-h oscillometric ABPM | Mean difference and SD; Bland–Altman; correlation coefficient | Complex procedures; not applicable to all devices or critical care | Several authors report manufacturer studies/fees | Italian Ministry of Health; NIH |
| Consensus | Cohen JB et al. | 2025 | NA | Cuffless BP devices | Scientific statement | Calibration by cuff and demographic approaches discussed | AHA statement; ISO 81060-2/3/7; IEEE 1708/1708a | Auscultation or intra-arterial monitoring | Mean ± SD; mean absolute difference | NR | Some authors report links/funding related to BP technologies | NIH; Apple; Microsoft; Australian and other funders |
| Regulatory document | FDA 510(k) | 2025 | NA | Aktiia G0 / Hilo cuffless wearable system | Regulatory review | NR | FDA 510(k) | NA | NR | Not peer-reviewed clinical study | NR | NR |
| Self-test / case study | Schutte AE | 2024 | 1 subject | Aktiia wristband; Biobeat chest patch; CART-I ring | 24-h self-monitoring including sleep and stress | Aktiia proprietary cuff; Biobeat/CART-I via iHealth cuff | NR | WatchBP O3 ABPM/HBPM reference | Average readings; p-values | n = 1; not generalizable | STRIDE BP co-chair; honoraria/advisor roles | NHMRC Investigator Grant |
| Systematic review/meta-analysis | Islam SMS et al. | 2022 | 16 studies; 974 participants | 15 wearable cuffless BP devices | Mixed lab/clinic | Device-dependent | ANSI/AAMI/ISO; ESH/ESH-IP; IEEE | Various: ABPM, mercury sphygmomanometer, others | Mean difference, SD, LOA; heterogeneity | High heterogeneity; early evidence | None declared | Australian National Heart Foundation; NHMRC fellowship |
| Narrative review | Pandit JA et al. | 2020 | NR | Cuffless BP generally | NR | Intermittent calibration with cuff highlighted | NR | Brachial cuff or arterial line (theoretical) | NR | NR | Patent/founder links reported | NIDDK |
| Narrative review | Yang E et al. | 2025 | NR | Multiple cuffless devices | International perspective | PWA/PPG + AI/ML; initial cuff or biometric calibration | ISO 81060-2; IEEE 1708; ISO 81060-3; ESH 2023 | Auscultatory sphygmomanometry; intra-arterial | Pressure difference thresholds | Lack of independent external validation | Author links with device companies | Microsoft; NIH |
| Interpretive review | Mukkamala R et al. | 2023 | NR; summarizes Aurora-BP project with 1125 participants | Microsoft Aurora Project / cuffless BP technologies | Interpretive review of large cuffless BP project | Emphasizes calibration, intended use, and post-calibration behavior | Contextual discussion of validation standards and regulatory implications | Aurora-BP study; cuff and ambulatory references as discussed | Narrative interpretation of study findings, physiology, and regulatory context | Secondary evidence; not a primary validation study | Several authors have cuffless BP and standards-related academic roles; consult article for details | NR |
| Review | Hua J et al. | 2024 | NR | Wearable cuffless BP | NA | Theoretical PTT/PWV models | NR | Cuff sphygmomanometer as general standard | NR | Engineering focus; accuracy affected by environment | Editorial-board relation reported; others none | Chinese national research funders |
| Review | Mukkamala R et al. | 2021 | NR | Cuffless BP devices | NR | Periodic calibration with conventional cuff | AAMI/BHS/ISO/IEEE | Manual or ambulatory auscultatory cuff device | SD, Bland–Altman, bootstrap, paired t-test, Pittman–Morgan | NR | Patents licensed to Digitouch Health and Samsung | US NIH |
| Validation study | Kuwabara M et al. | 2019 | 85 subjects per device | Omron HEM-6410T-ZM/ZL | Clinical | NR | ANSI/AAMI/ISO 81060-2:2013 | Mercury sphygmomanometer | Mean difference ± SD; ISO criteria; Bland–Altman | Wrist-position sensitivity; controlled conditions | Omron employees | Jichi Medical University; Omron Healthcare |
| Comparative study | Kario K et al. | 2020 | 50 patients; 66.1 ± 10.8 years | Omron HeartGuide | Clinic and outpatient | Position sensor at heart level | ANSI/AAMI/ISO 81060-2:2013 | A&D TM-2441 ABPM | t-test; mixed-effects models | Order effect in consecutive same-arm measurements | Consultancies/scholarships involving Omron/A&D | Omron Healthcare |
| Feasibility/reliability study | Groppelli A et al. | 2022 | 81 patients; 61 ± 19 years | Omron HeartGuide 6410T | Clinical tilt-table testing | Heart-level position check before inflation | ANSI/AAMI/ISO 81060-2:2013 | Finometer; Task Force monitor; AND UA-651 | Lin/Pearson concordance; Bland–Altman | Long measurement time; error signals | None declared | Italian Ministry of Health |
| Comparative study | Vaseekaran M et al. | 2023 | 159 patients; 64.3 ± 16.1 years | Omron HeartGuide; Smart Wear TV962SM | Cardiology ward/outpatient | Heart-level position instructions | Riva-Rocci method | Auscultatory sphygmomanometry; conventional ABPM | ICC, Bland–Altman, LOESS | Small sample; no blinding/randomization | None declared | No external funding |
| Validation study | Zhang W et al. | 2022 | 85 adults analyzed | HUAWEI WATCH D | Clinical validation | NR | AAMI/ESH/ISO Universal Standard | Two mercury sphygmomanometers in Y configuration | Mean ± SD; criteria 1 and 2; Bland–Altman | Excluded wrist circumference > 20 cm and other cardiac conditions | Fees from industry reported by one author | HUAWEI |
| Validation study | Zhou Y et al. | 2025 | 95 recruited; 85 analyzed; 35 for 24-h | HUAWEI WATCH D2 | Rest + ambulatory | NR | AAMI/ESH/ISO Universal Standard + AMD 1:2020 | A&D TM-2430 ABPM and observers | Mean ± SD; Universal Standard criteria | NR | Industry fees reported by one author | HUAWEI; Chinese public funders |
| Validation study | Wang L et al. | 2022 | 85 subjects; 21–85 years | HUAWEI WATCH BP function | Clinical | Pulse-wave analysis during inflation; unspecified algorithm | ANSI/AAMI/ISO 81060-2:2018 | Calibrated mercury sphygmomanometer | Mean BP difference; SD; Bland–Altman | Validated only on left wrist at heart level | Some authors employed by Huawei | West China Hospital-Huawei; Huawei Device Co. |
| Validation study | Yi L et al. | 2022 | 361 subjects | HUAWEI WATCH D | Clinical rest/outpatient/circadian | PPG technology; calibration twice/day | ISO 81060-2:2018; YY0670-2008 | Mercury sphygmomanometer; automatic oscillometric | Confusion matrix; Bland–Altman | High invalid-data rate in circadian study | Several Huawei employees | China Key National R&D Program |
| Validation study | Moon JH et al. | 2020 | 35 adults; 57.1 ± 17.9 years | InBodyWATCH | Clinical | Neural-network model with 3 reference BP sets | ESH 2010; IEEE 1708 | Manual sphygmomanometer | Pearson r; Bland–Altman; RMSE | Radial vs forearm measurement differences | Two authors employed by InBody | World Class 300; InBody |
| Cross-sectional validation | Falter M et al. | 2022 | 40 patients | Samsung Galaxy Watch Active2 | Clinical simultaneous with 24-h ABPM | 3 reference measurements | Taffé method; ISO 81060-2:2018 | Mobil-O-Graph ABPM; Omron M2 | Sensitivity, specificity, PPV/NPV, ROC, Bland–Altman | Systematic bias: overestimates low BP, underestimates high BP | None declared | CoroPrevention; FWO; Italian Ministry of Health |
| Prospective observational | Han M et al. | 2023 | 760 participants | Samsung Galaxy Watch | Real-world | Self-calibration with arm monitor | AAMI/ESH/ISO Universal Standard | Validated automatic arm monitor | Mixed-effects model; logistic regression; ANOVA | Not a formal clinical trial; unrestricted behavior | Honoraria/grants from device companies by one author | Korean Hypertension Society |
| Feasibility study | Lee Y et al. | 2025 | 896 participants | Samsung Galaxy Watch6 | Daily-life monitoring campaign | Initial and weekly recalibration with cuff | Korean Society of Hypertension standards | Validated automatic arm monitor | t-test, ANOVA, Tukey, chi-square | May miss clinical variability; calibration error | Honoraria from SkyLab by one author | National Research Foundation of Korea |
| Validation study | Ahn JH et al. | 2021 | 56 Parkinson disease patients; 168 pairs | Samsung SM-R850 smartwatch | Clinical neurology; seated/rest | Calibrated with aneroid sphygmomanometer | ISO BP validation criteria | Aneroid sphygmomanometer | Criteria 1 and 2; Bland–Altman; correlations | Excluded severe tremor/arrhythmia; single clinical setting | None declared | NR |
| Validation study | Liu YH et al. | 2025 | 35 acute ischemic stroke patients | ASUS VivoWatch SP | Hospital neurology unit | First reference BP pair entered into app | ANSI/AAMI/ISO 81060-2:2018 | Mercury sphygmomanometer | Criteria 1 and 2; Bland–Altman | Small sample; rest only | None declared | E-DA Hospital |
| Large-scale validation | Liu ZD et al. | 2023 | 3077 participants | Huawei smartwatch prototype | Outpatient setting | Baseline BP average of first day | ANSI/AAMI/ISO; IEEE 1708; BHS | Two-observer auscultation | ME, SDE, MAE, CPE | Unbalanced BP distribution; 1-month robustness only | Huawei-linked authors | Chinese public research funders |
| Validation study | Vybornova A et al. | 2021 | 86 participants | Aktiia Bracelet | Outpatient | Subject-dependent parameters from first two reference measurements | Extended ISO 81060-2:2013 | Double-blind auscultation | Mean error, SD, ISO criteria, Bland–Altman, t-test | No complete standard for optical devices | Aktiia employees/consultants | Aktiia SA |
| Validation study | Solà J et al. | 2021 | 91 patients | Aktiia Bracelet | Daily life; sitting/lying/standing | Oscillometric arm cuff initialization | Extended ISO 81060-2 protocol | Double-blind manual auscultation | Mean/SD differences; correlations; Bland–Altman | SBP requirement deviation; non-standard-position uncertainty | Aktiia employees/consultants | NR |
| Prospective study | Almeida TP et al. | 2023 | 52 patients | Aktiia cuffless monitor | Cardiac rehabilitation; outpatient | Oscillometric initialization | NR | Dyasis 3 ABPM | Mean ± SD; 95% CI; t-test; correlation; Bland–Altman | Preliminary data; mostly normotensive | Aktiia employees/consultants | NR |
| Tracking changes study | Tan I et al. | 2023 | 41 participants; 80% hypertensive; 3 medication-uptitration cases | Aktiia wrist wearable | 6–12 days continuous; ABPM/HBPM comparisons | Device-specific initialization/calibration | NR | 24-h ABPM; cuff-based HBPM | Mean differences; CI; dipping; medication-response comparison | Did not accurately track nighttime dipping or medication-induced changes | NR in abstract/source consulted | NR |
| Large independent validation/dataset study | Mieloszyk R et al. | 2022 | 1125 participants with usable recordings | Microsoft Aurora-BP; wearable tonometry, PPG, ECG, and accelerometry | Controlled and ambulatory settings | Reference-dependent calibration and posture-sensitive testing; device/project-specific procedures | Device/project-specific validation; comparisons contextualized with cuffless BP standards | Dual-observer auscultation and oscillometric ABPM references | BP-estimation error by signal type, reference type, posture relative to calibration, and controlled vs ambulatory condition | Performance strongly affected by reference type, posture, calibration, and testing condition; tonometry informative only when signal quality is adequate | NR in extracted source | Microsoft Research / project-specific funding context |
| Prospective observational | Almeida TP et al. | 2025 | 54 patients | Aktiia cuffless monitor | 24-h/day/night vs ABPM | Oscillometric initialization | NR | Dyasis 3 ABPM | Mean ± SD; 95% CI; t-test; correlation; Bland–Altman; ROC/AUC | Mostly normotensive; not designed for rehabilitation effect | Aktiia employees/consultants | NA |
| Cross-sectional study | van Vliet M et al. | 2024 | 97 patients | CardioWatch 287-2 wristband | Cardiac catheterization | Three cuff measurements + demographics | AAMI/ESH/ISO Universal Standard | Invasive subclavian BP | Mean error; SD; % within 5/10/15; Bland–Altman | Single center; limited patients | Consultant at Corsano Health | Corsano Health BV |
| Comparative validation | Nachman D et al. | 2020 | 1057 subjects | Biobeat BB-613WP PPG device | Public BP screening stations | Baseline cuff value entered for PPG device; 3-month validity per device | ISO 81060-2:2013 | Welch Allyn DuraShock cuff sphygmomanometer | Mean ± SD difference; ICC; agreement; ROC/Kappa | No double-observer reference; rounding to nearest 5 mmHg; pragmatic setting | Biobeat employees among authors | NR |
| Clinical trial | Nachman D et al. | 2021 | 28 subjects; 2381 paired measurements | Biobeat Wrist-monitor BB-613WP | 24-h ambulatory monitoring | Average of 3 cuff-based baseline measurements; calibration once at start | NR; equivalence testing | CONTEC ABPM50 oscillometric ABPM | Bland–Altman; regression; ROC/AUC; TOST | Small sample; few hypertensive/elderly/obese/extreme-BP subjects; no bilateral BP pretest | Biobeat employees; devices supplied free | None |
| Validation study | Kim S et al. | 2018 | 34 uncontrolled hypertensive patients | LG watch-style magnetoplethysmography BP monitor | Clinical | Baseline BP and pulse-wave morphological index | IEEE 1708-2014; FDA | Auscultatory sphygmomanometer; Omron HEM-7121 | ICC; Bland–Altman; mean ± SD; MAD | Hypertensive-only; non-simultaneous reference; controlled setting | LG employees | LG Electronics |
| Real-life validation | Schoot TS et al. | 2016 | 37 volunteers | Checkme Health Monitor | Clinical / real-life | Personal profile + reference SBP | ESH-IP | Welch Allyn Vital Signs Monitor 300 | Differences > 5/10/15; correlation; Bland–Altman; t-test | No uniform protocol; no DBP; calibration failures | None declared | NR |
| Validation study | Boubouchairopoulou N et al. | 2017 | 85 subjects | Freescan | Hypertension clinic self-measurement | Individual calibration using validated arm monitor | ANSI/AAMI/ISO 2013 | Mercury sphygmomanometer | Mean difference; inter-subject SD | Prototype; no long-term stability without recalibration | Maisense links | Maisense Inc. |
| Diagnostic accuracy | Kyriakoulis KG et al. | 2019 | 136 elderly hypertensive subjects | Freescan AF detection | Outpatient clinic | Individual mercury calibration | ANSI/AAMI/ISO 2013 | Validated arm cuff monitor | Specificity; sensitivity; diagnostic accuracy | High error/instability failure rate | Consulting fees from Maisense by one author | Maisense; University of Athens research grants |
| Pilot feasibility | Ogink PAM et al. | 2019 | 11 hypertensive patients | Checkme Pro Health Monitor | Outpatient/home | Clinically obtained SBP value | CE marking; FDA 510(k) | Welch Allyn automatic and patient-owned monitors | Intra-user correlation; SUS; Spearman | Small sample; short follow-up; medication influence | None declared | NR |
| Validation study | van Helmond N et al. | 2019 | 85 analyzed | Everlast smartwatch; BodiMetrics Performance Monitor | Hospital pre-admission testing | BodiMetrics initialized with SBP; Everlast no calibration | ANSI/AAMI/ISO 2013; BHS | Cardiocap 5 | MAD, SD; Bland–Altman; Pearson/Spearman; RMSE | Not accurate for pressure changes or long post-calibration interval | RTM Vital Signs founder/owner among authors | Not funded |
| Validation study | Hahnen C et al. | 2020 | Adults | Everlast smartwatch; BodiMetrics portable monitor | Hospital testing center | BodiMetrics initialized with SBP; Everlast no calibration | ANSI/AAMI/ISO 2013; BHS | Cardiocap 5 | Bland–Altman; Pearson/Spearman; RMSE; MAD | Automatic monitor not mercury; no induced BP changes | RTM Vital Signs founder/owner among authors | No specific funding |
| Validation study | Bilo G et al. | 2015 | 33 adults | SOMNOtouch-NIBP | Clinical/laboratory | Initial brachial cuff measurement | ESH-IP 2010 revision | Mercury sphygmomanometer, two observers | Absolute differences; correlation | Reduced accuracy at very high pressures; DBP underestimation; altitude bias | Speaker fees from Somnomedics | Somnomedics GmbH |
| ABPM agreement study | Nyvad J et al. | 2021 | 51 hypertensive patients | SOMNOtouch-NIBP | 24-h outpatient ABPM | Initial single brachial cuff measurement | NICE; ESH/ISO | Spacelabs 90217 oscillometric | 95% LOA; Pearson; linear regression | Poor agreement; nighttime overestimation; low dipping detection | None declared | Aarhus University Hospital |
| Validation/acceptability | Islam SMS et al. | 2019 | 20 healthy volunteers | TMART T2 wrist-worn cuffless | 24-h outpatient + 7-day home | No prior calibration per device | IEEE 1708-2014; ANSI/AAMI/ISO | A&D TM-2430 ABPM; Omron HEM-7121 | Pearson; Bland–Altman; MAD; MAPD; Mann–Whitney U | Poor synchronization; young healthy sample; limited sensor specs | None declared | IPAN; Deakin University |
| Prototype validation | Carek AM et al. | 2017 | 13 young healthy subjects | SeismoWatch | Laboratory | Subject-specific K1/K2 via regression | FDA error limits | ccNexfin | RMSE; R; Bland–Altman; p-values | Young healthy only; requires maneuver; artifact-sensitive; cuff calibration | NR | NIH |
| Pilot study | Kachel E et al. | 2021 | 10 post-cardiac surgery patients | Biobeat PPG wrist + chest | ICU post cardiac surgery | Mean of first 3 arterial-line measurements | ESH, BHS, IEEE 1708 post hoc | Invasive arterial line | Bland–Altman; Pearson; bias; LOA; sensitivity/specificity | Small sample; post-op setting | Biobeat advisor/employees | Israeli Ministry of Science; Tel Aviv University |
| Validation study | Jakes A et al. | 2021 | 45 pregnant women | BPro radial pulse waveform device | Pregnancy/gestational hypertensive disorders | Manual arm measurement calibration | AAMI/ESH/ISO | Mercury sphygmomanometer | Mean differences ± SD; % within 5/10/15; Bland–Altman | Few/no obese participants | None declared | NR |
| Prototype validation | Nabeel PM et al. | 2018 | 83 subjects | Carotid compliance probe | Clinical/laboratory | NR | NR | Brachial cuff SBP/DBP | Pearson r; Bland–Altman; ROC; absolute error | Specialist setup; carotid practicality | NR | SERB/DST Government of India |
| Feasibility study | Raj KV et al. | 2022 | 8 male subjects | Carotid ultrasound model-based BP | Laboratory intervention | NR | NR | Human NIBP Nano | Mean differences; paired/Welch tests; Bland–Altman; correlation; MAE | Very small male-only sample | None declared | NR |
| Validation study | Yamanaka S et al. | 2021 | Diverse age/BP population | ML-based cuffless BP estimation | Clinical validation | Calibration-free | ISO 81060-1:2007 | Mercury sphygmomanometer, two observers | Mean error; SD | Limited sample size; poor generalizability | Author employed by Connect Inc. | Panasonic; hospital; Connect Inc. |
| Validation study | López-Ortiz S et al. | 2025 | 100 participants; 1000 pairs | KC08 low-cost smartwatch | Controlled validation | NR | NR | Reference device for BP; reference devices for HR/SpO2 | Significance tests; variability; reliability; Bland–Altman/heteroscedasticity reported | Not suitable for daily clinical practice for BP; poor reliability except HR | NR in source consulted | NR |
| First-in-human pivotal | Kim J et al. | 2024 | 89 subjects | CART-I Plus smart ring | Clinical care | Two auscultatory reference sets | ISO 81060-2:2018; Korean FDA | Auscultatory sphygmomanometer | Mean difference and SD; regression; Bland–Altman | Small sample; limited ethnic representation; site variation | None declared | Samsung Medical Center |
| Feasibility/effectiveness | Lee H et al. | 2024 | 33 participants | CART-I Plus ring | Out-of-office monitoring | Omron HEM-7080 calibration/reference | ESH; ISO 81060-2:2018 | Conventional ABPM | Mean error and SD; Pearson; Bland–Altman | Small sample; calibration differs from recent recommendations | None declared | Sky Labs Inc. |
| Continuous validation | Kwon Y et al. | 2022 | 34 ICU patients | CareTaker finger sensor | Surgical/trauma ICU | 15-s startup window with patient-specific constants | ANSI/AAMI/ISO 81060-2:2013 | Radial intra-arterial catheter | Pearson; bias; precision; Cronbach alpha | ICU population; device-specific constraints | CareTaker employee among authors | CareTaker Medical Inc. |
| Perioperative validation study | Gratz I et al. | 2017 | 24 adult patients undergoing major intra-abdominal surgery | CareTaker continuous non-invasive finger cuff | Major abdominal surgery / perioperative monitoring | Arterial-line BP used during initial startup/calibration procedure; device-specific algorithm | ANSI/AAMI/ISO 81060-2:2013-related criteria | Radial intra-arterial catheter | Pearson correlation; Bland–Altman; polar plot; four-quadrant trend analysis | Pilot sample; limited severe hypotension; 30-min analysis window; recalibration may be needed with major hemodynamic or arterial-stiffness changes | Device provided by Empirical Technologies Corporation; consult article for details | Unfunded study; NIH training support for one author |
| Perioperative validation study | Gratz I et al. | 2021 | 41 major abdominal surgery patients | Next-generation CareTaker continuous physiological monitor | Abdominal surgery / perioperative monitoring | Device-specific calibration/startup procedure with finger cuff on contralateral middle finger | ANSI/AAMI/ISO 81060-2:2019 | Invasive intra-arterial pressure and clinical-grade oscillometric cuff | Pearson correlation; Bland–Altman; four-quadrant trend analysis | Perioperative setting; manufacturer-linked device; external generalizability uncertain | NR in extracted source | NR in extracted source |
| Cardiac catheterization validation/tracking study | Kwon Y et al. | 2022 | 47 patients undergoing cardiac catheterization; 31369 beats analyzed | CareTaker physiological monitor | Cardiac catheterization | Device-specific beat-to-beat recording; central aortic comparison; calibration details reported in full article | NR / device-specific validation framework | Invasive central aortic BP | Pearson correlation; Bland–Altman; four-quadrant concordance for beat-to-beat trends | Central aortic comparison; absolute peripheral-to-central translation uncertain; setting-specific evidence | NR in extracted source | NR in extracted source |
| Pediatric surgical validation study | Boretsky KR et al. | 2025 | Children aged 2–17 years undergoing major surgery | VitalStream continuous non-invasive BP monitor | Pediatric surgery | Device-specific VitalStream calibration/startup procedure | ISO 81060-3:2022 elements | Invasive intraarterial pressure | Agreement with invasive pressure; correlation/concordance and ISO-related performance metrics | Pediatric surgical setting; generalizability outside major surgery and artifact conditions remains uncertain | NR in extracted source | NR in extracted source |
| Pragmatic validation/usability study | Hamilton LD et al. | 2024 | 25 ED patients, 25 ICU patients; 13 ED nurses and 12 ICU nurses for usability | VitalStream / CareTaker finger-cuff continuous BP monitor | Level-1 trauma center emergency department and ICU | ED: automatic calibration every 7 min; ICU: randomized manual vs automatic calibration; manual calibration used arterial-line BP at session start without subsequent recalibration; automatic calibration included recalibration every 7 min | AAMI 2008; AAMI/ESH/ISO 2019 consensus criteria | ED: manual brachial cuff/auscultation; ICU: arterial line | Bland–Altman bias/LOA; bootstrapped paired differences; % within 10 mmHg; Pearson correlation; Lin’s concordance; SUS; NPS | Did not meet AAMI/AAMI-ESH-ISO criteria; SD exceeded 8 mmHg in all comparisons; fewer than 85% of paired differences within 10 mmHg; single hospital; movement artifact; possible cuff-fit and low-perfusion limitations; practical analysis without postprocessing artifact removal | Conflicts of interest: none reported | CareTaker Medical provided financial support but was not involved in study design, data collection, analysis, or interpretation |

***Legend:*** *BP, blood pressure; SBP, systolic blood pressure; DBP, diastolic blood pressure; ABPM, ambulatory blood pressure monitoring; HBPM, home blood pressure monitoring; PPG, photoplethysmography; PTT, pulse transit time; PAT, pulse arrival time; PWA, pulse wave analysis; LOA, limits of agreement; MAD, mean absolute difference; MAPD, mean absolute percent difference; RMSE, root mean square error; NR, not reported; NA, not applicable.*

**Supplementary Material S2.** Device map.

| **Device / family** | **Type** | **Form factor** | **Technology** | **Key evidence IDs** | **Highest validation evidence found** | **Key gaps** |
| --- | --- | --- | --- | --- | --- | --- |
| Omron HEM-6410T / HeartGuide | Wearable | Watch-type wrist, cuff-based | Oscillometry with mini cuff | P01–P04 | ANSI/AAMI/ISO validation and clinical/ABPM comparisons | Ambulatory/dynamic performance, nighttime use, long-term adherence |
| HUAWEI WATCH D / D2 | Wearable | Watch-type wrist, cuff-based | Oscillometric wrist cuff | P05–P08 | Universal Standard validation; rest and ambulatory assessment | Independent real-world ABPM comparisons; broader populations; arrhythmias |
| Samsung Galaxy Watch family | Wearable | Smartwatch | PPG/PWA calibrated algorithm | P10–P13 | Real-world feasibility; clinical validation including Parkinson disease | Calibration stability, treatment/exercise/night dipping, high/low BP bias |
| Aktiia / Hilo / G0 | Wearable | Wrist bracelet | PPG + pulse-wave analysis/cloud algorithms | P16–P20; G07; EC01 | Extended ISO-based validation; ABPM comparisons; FDA 510(k) | Independent long-term outcomes; nighttime dipping and medication-response tracking; drift |
| Microsoft Aurora-BP | Wearable/portable research platform | Wrist/arm wearable sensors and ambulatory acquisition setup | Radial tonometry, PPG, ECG, accelerometry, and multimodal models | Mieloszyk 2022; Mukkamala 2023 | Large independent controlled/ambulatory dataset comparing signal modalities and reference types | Performance strongly affected by reference, posture relative to calibration, ambulatory conditions, and signal quality; not a simple endorsement of any single cuffless technology |
| Biobeat BB-613WP / Wrist-monitor | Wearable | Wrist/skin-attached device; chest patch in ICU study | Reflective PPG + PWTT/PWA | P22; P23; P35 | Large screening-station comparison; 24-h ABPM study; arterial-line ICU pilot | Subgroups with extreme BP, older/obese/hypertensive samples; independent replication |
| CardioWatch 287-2 / Corsano | Wearable | Wristband | Continuous PPG-based algorithm | P21 | Compared with invasive BP under Universal Standard framework | ESH multi-test validation, movement/exercise/day-night/recalibration |
| InBodyWATCH | Wearable | Wristwatch | Cuffless NN model | P09 | Validation vs manual sphygmomanometer | External validation, ambulatory use, calibration stability |
| KC08 smartwatch | Wearable | Low-cost smartwatch | Commercial smartwatch; likely PPG-based | P40 | 1000-pair validation against reference device | Poor reliability for BP; not suitable for daily clinical practice |
| CART-I Plus | Wearable | Smart ring | Finger PPG cuffless BP | P41; P42 | First-in-human pivotal study and ABPM comparison | Motion/vasoconstriction, diverse populations, recalibration tests |
| CareTaker / VitalStream | Wearable/portable | Finger cuff/finger sensor with wrist-worn unit | Pulse Decomposition Analysis | Kwon 2022 ICU; Gratz 2017/2021; Kwon 2022 central aortic; Boretsky 2025; Hamilton 2024 | Compared with invasive radial arterial catheter, invasive central aortic pressure, perioperative arterial catheter, pediatric intra-arterial pressure, ED brachial cuff, and ICU arterial line | Evidence includes both favorable controlled/postprocessed studies and pragmatic negative ED/ICU findings; remaining gaps include artifact handling, low-perfusion states, calibration/recalibration reporting, pediatric and acute-care generalizability, and intended-use-specific validation. |
| SOMNOtouch-NIBP | Wearable/portable | ABPM-style wearable | Cuffless continuous, PTT-based | P31; P32 | ESH-IP validation; later poor agreement with 24-h ABPM | Reconciling discrepant evidence; drift; arrhythmias; nighttime performance |
| SeismoWatch | Wearable prototype | Watch + sternum maneuver | SCG + PPG → PTT | P34 | Accuracy/repeatability in young adults | Daily-life usability, diverse populations |
| TMART T2 | Wearable | Wrist-worn | Device-specific cuffless BP | P33 | Compared with ABPM and HBPM; acceptability assessed | Standard validation, long-term drift, subgroup performance |
| Checkme / Checkme Pro | Portable cuffless | Pocket device | PTT-based BP | P25; P28 | Real-life evaluation and mixed-method feasibility | Formal protocol accuracy, calibration stability |
| Freescan | Portable cuffless | Pocket radial device | Electrodes + force sensor over radial pulse | P26; P27 | Clinical validation and AF-screening diagnostic study | Independent BP validation; arrhythmia effects |
| Everlast / BodiMetrics | Wearable/portable | Smartwatch + portable monitor | Proprietary cuffless approaches | P29; P30 | Independent validation showed insufficient accuracy | General lesson: require independent validation before clinical use |
| BPro | Wearable | Radial pulse waveform device | Tonometer/radial pulse waveform | P36 | Validation in pregnancy and gestational hypertensive disorders | Obesity, broader pregnancy populations, ambulatory conditions |
| Carotid ultrasound/compliance prototypes | Portable prototype | Carotid probe / ultrasound setup | Model-based calibration-free BP | P37; P38 | Feasibility/experimental validation | Wearability, operator dependence, invasive-reference validation |
| ML calibration-free device/prototype | Prototype | Unspecified system | Population-based ML cuffless BP estimation | P39 | Clinical validation with mercury sphygmomanometer | Generalizability, transparency, external validation |

# Appendix 1. Search strategies

## 1. PubMed/MEDLINE

("Blood Pressure Determination"[Mesh] OR "Blood Pressure Monitors"[Mesh] OR "Blood Pressure Monitoring, Ambulatory"[Mesh] OR "blood pressure monitoring"[tiab] OR "blood pressure measurement"[tiab] OR "blood pressure estimation"[tiab]) AND (cuffless[tiab] OR "cuff-less"[tiab] OR "cuff free"[tiab] OR "Wearable Electronic Devices"[Mesh] OR wearable*[tiab] OR smartwatch*[tiab] OR "smart watch"[tiab] OR "wrist-worn"[tiab] OR wristband*[tiab] OR "smart ring"[tiab] OR patch*[tiab] OR smartphone*[tiab] OR "smartphone-based"[tiab]) AND (photoplethysmograph*[tiab] OR PPG[tiab] OR electrocardiograph*[tiab] OR ECG[tiab] OR "pulse transit time"[tiab] OR PTT[tiab] OR "pulse wave velocity"[tiab] OR PWV[tiab] OR "pulse arrival time"[tiab] OR PAT[tiab] OR bioimpedance[tiab] OR tonometr*[tiab] OR ballistocardiograph*[tiab] OR BCG[tiab] OR "remote photoplethysmography"[tiab] OR rPPG[tiab]) NOT (ocular[tiab] OR intraocular[tiab] OR intracranial[tiab] OR pulmonary[tiab] OR portal[tiab]) NOT (animals[mh] NOT humans[mh])

## 2. Scopus

TITLE-ABS-KEY(("blood pressure monitoring" OR "blood pressure measurement" OR "blood pressure estimation") AND (cuffless OR "cuff-less" OR "cuff free" OR wearable* OR smartwatch* OR "smart watch" OR "wrist-worn" OR wristband* OR "smart ring" OR patch* OR smartphone* OR "smartphone-based") AND (photoplethysmograph* OR PPG OR electrocardiograph* OR ECG OR "pulse transit time" OR PTT OR "pulse wave velocity" OR PWV OR "pulse arrival time" OR PAT OR bioimpedance OR tonometr* OR ballistocardiograph* OR BCG OR "remote photoplethysmography" OR rPPG)) AND NOT TITLE-ABS-KEY(ocular OR intraocular OR intracranial OR pulmonary OR portal)

## 3. Embase

('blood pressure monitoring':ti,ab,kw OR 'blood pressure measurement':ti,ab,kw OR 'blood pressure estimation':ti,ab,kw) AND (cuffless:ti,ab,kw OR 'cuff-less':ti,ab,kw OR 'cuff free':ti,ab,kw OR wearable*:ti,ab,kw OR smartwatch*:ti,ab,kw OR 'smart watch':ti,ab,kw OR 'wrist-worn':ti,ab,kw OR wristband*:ti,ab,kw OR 'smart ring':ti,ab,kw OR patch*:ti,ab,kw OR smartphone*:ti,ab,kw OR 'smartphone-based':ti,ab,kw) AND (photoplethysmograph*:ti,ab,kw OR PPG:ti,ab,kw OR electrocardiograph*:ti,ab,kw OR ECG:ti,ab,kw OR 'pulse transit time':ti,ab,kw OR PTT:ti,ab,kw OR 'pulse wave velocity':ti,ab,kw OR PWV:ti,ab,kw OR 'pulse arrival time':ti,ab,kw OR PAT:ti,ab,kw OR bioimpedance:ti,ab,kw OR tonometr*:ti,ab,kw OR ballistocardiograph*:ti,ab,kw OR BCG:ti,ab,kw OR 'remote photoplethysmography':ti,ab,kw OR rPPG:ti,ab,kw) NOT (ocular:ti,ab,kw OR intraocular:ti,ab,kw OR intracranial:ti,ab,kw OR pulmonary:ti,ab,kw OR portal:ti,ab,kw)

## 4. Web of Science

TS=(("blood pressure monitoring" OR "blood pressure measurement" OR "blood pressure estimation") AND (cuffless OR "cuff-less" OR "cuff free" OR wearable* OR smartwatch* OR "smart watch" OR "wrist-worn" OR wristband* OR "smart ring" OR patch* OR smartphone* OR "smartphone-based") AND (photoplethysmograph* OR PPG OR electrocardiograph* OR ECG OR "pulse transit time" OR PTT OR "pulse wave velocity" OR PWV OR "pulse arrival time" OR PAT OR bioimpedance OR tonometr* OR ballistocardiograph* OR BCG OR "remote photoplethysmography" OR rPPG)) NOT TS=(ocular OR intraocular OR intracranial OR pulmonary OR portal)

## Complementary targeted searches

## HeartGuide; HEM-6410T; HUAWEI WATCH D; HUAWEI WATCH D2; Aktiia; Hilo; SOMNOtouch-NIBP; SeismoWatch; CART-I Plus; Freescan; Checkme; BioBeat; CareTaker; VitalStream; Aurora-BP; Microsoft Aurora; BPro; Samsung Galaxy Watch; SM-R850; KC08.

# Related supplementary file

The completed PRISMA-ScR checklist is provided separately as Supplementary Material 2.
