# Supplementary material for: Wearable, cuffless, and portable devices for blood pressure monitoring (2015–2025): a scoping review of technologies, calibration, clinical validation, and regulatory frameworks
Source: Front Digit Health. 2026 Aug 12;8:1870156. doi: 10.3389/fdgth.2026.1870156 (PMC13506816; doi:10.3389/fdgth.2026.1870156)
Supplement: Supplementary file 2 [file Supplementaryfile2.docx]

Supplementary Material 2 — PRISMA-ScR Checklist

**Manuscript:** *Wearable, cuffless, and portable devices for blood pressure monitoring (2015–2025): a scoping review of technologies, calibration, clinical validation, and regulatory frameworks.*

*This checklist documents the location in the manuscript or supplementary material where each of the 22 PRISMA-ScR items is reported. Items not applicable to a scoping review (e.g., critical appraisal) are flagged accordingly.*

| **Section** | **Item** | **PRISMA-ScR checklist item** | **Reported location in manuscript** |
| --- | --- | --- | --- |
| **Title** | 1 | Identifies the report as a scoping review. | Title |
| **Abstract** | 2 | Includes background, objectives, eligibility criteria, sources, extraction, results, and conclusions. | Abstract (updated evidence counts and synthesis summary) |
| **Introduction** | 3 | Rationale for the review and context of prior knowledge. | Introduction |
| **Introduction** | 4 | Explicit objectives with PCC or other key elements. | Introduction (final paragraph) |
| **Methods** | 5 | Protocol and registration: indicate existence/access. | Methods 2.1 Design and reporting guidance |
| **Methods** | 6 | Eligibility criteria. | Methods 2.2 PCC framework and eligibility criteria |
| **Methods** | 7 | Information sources and search date. | Methods 2.3 Information sources and search strategy (updated targeted device/project-name searches) |
| **Methods** | 8 | Reproducible search strategy. | Appendix 1 (Supplementary Material; updated complementary targeted searches) |
| **Methods** | 9 | Source selection process. | Methods 2.4 Reference management and source selection |
| **Methods** | 10 | Charting/data extraction process. | Methods 2.5 Data extraction and synthesis |
| **Methods** | 11 | Extracted variables. | Methods 2.5 and Supplementary Table S1 |
| **Methods** | 12 | Critical appraisal of individual sources, if performed. | Not performed; justified in Methods 2.1 |
| **Methods** | 13 | Synthesis method. | Methods 2.5 (descriptive synthesis) |
| **Results** | 14 | Numbers of sources screened, assessed, and included; ideally a flow diagram. | Results 3.1 and Figure 1 (updated PRISMA-ScR flow diagram; n = 63) |
| **Results** | 15 | Characteristics of included sources. | Results 3.2, Table 1, and Supplementary Table S1 (updated characteristics; n = 63) |
| **Results** | 16 | Critical appraisal within sources, if performed. | Not applicable (not performed) |
| **Results** | 17 | Relevant data from each source. | Supplementary Table S1 (updated extraction, including additional Aurora and CareTaker/VitalStream sources) |
| **Results** | 18 | Synthesis of results aligned with objectives. | Results 3.2-3.6 (updated technology-category synthesis) |
| **Discussion** | 19 | Summary of evidence and relevance to objectives, populations, and stakeholders. | Discussion (summary of evidence, intended-use interpretation, calibration, algorithms, and comparator heterogeneity) |
| **Discussion** | 20 | Limitations of the scoping review process. | Discussion (limitations of scoping-review process and evidence comparability) |
| **Discussion** | 21 | General interpretation, implications, and future directions. | Discussion and Conclusions (future directions and minimum reporting framework) |
| **Funding** | 22 | Funding sources for the review and role of funders. | Funding |

**Abbreviations:** PCC, Population–Concept–Context; PRISMA-ScR, Preferred Reporting Items for Systematic Reviews and Meta-Analyses extension for Scoping Reviews.
